# Costs along the service cascades for HIV testing and counselling and prevention of mother-to-child transmission

**DOI:** 10.1097/QAD.0000000000001208

**Published:** 2016-09-28

**Authors:** Sergio Bautista-Arredondo, Sandra G. Sosa-Rubí, Marjorie Opuni, David Contreras-Loya, Ada Kwan, Claire Chaumont, Abson Chompolola, Jeanine Condo, Omar Galárraga, Neil Martinson, Felix Masiye, Sabin Nsanzimana, Ivan Ochoa-Moreno, Richard Wamai, Joseph Wang’ombe

**Affiliations:** a National Institute of Public Health (INSP), Division of Health Economics, Cuernavaca, Mexico; b UNAIDS, Geneva, Switzerland; c Division of Economics, University of Zambia, Lusaka, Zambia; d National University of Rwanda, School of Public Health, Kigali, Rwanda; e Brown University, Providence, Rhode Island, USA; f Perinatal HIV Research Unit, University of the Witwatersrand, Johannesburg, South Africa; g Rwanda Biomedical Center, Kigali, Rwanda; h Northeastern University, Boston, Massachusetts, USA; i University of Nairobi, School of Public Health, Nairobi, Kenya.

**Keywords:** Africa, costs, economics, efficiency, HIV, HIV testing and counselling, prevention of mother-to-child transmission

## Abstract

We estimate facility-level average annual costs per client along the HIV testing and counselling (HTC) and prevention of mother-to-child transmission (PMTCT) service cascades.

**Design:**

Data collected covered the period 2011–2012 in 230 HTC and 212 PMTCT facilities in Kenya, Rwanda, South Africa, and Zambia.

**Methods:**

Input quantities and unit prices were collected, as were output data. Annual economic costs were estimated from the service providers’ perspective using micro-costing. Average annual costs per client in 2013 United States dollars (US$) were estimated along the service cascades.

**Results:**

For HTC, average cost per client tested ranged from US$5 (SD US$7) in Rwanda to US$31 (SD US$24) in South Africa, whereas average cost per client diagnosed as HIV-positive ranged from US$122 (SD US$119) in Zambia to US$1367 (SD US$2093) in Rwanda. For PMTCT, average cost per client tested ranged from US$18 (SD US$20) in Rwanda to US$89 (SD US$56) in South Africa; average cost per client diagnosed as HIV-positive ranged from US$567 (SD US$417) in Zambia to US$2021 (SD US$3210) in Rwanda; average cost per client on antiretroviral prophylaxis ranged from US$704 (SD US$610) in South Africa to US$2314 (SD US$3204) in Rwanda; and average cost per infant on nevirapine ranged from US$888 (SD US$884) in South Africa to US$2359 (SD US$3257) in Rwanda.

**Conclusion:**

We found important differences in unit costs along the HTC and PMTCT service cascades within and between countries suggesting that more efficient delivery of these services is possible.

## Introduction

After a decade of increases in financing for HIV services in low-income and middle-income countries (LMICs), funding has levelled off [1]. In this context, to scale up HIV services, countries must optimize use of available funds [2–4]. Empirical data on the per person cost of HIV services are critical to making better use of resources; they can be used to assess the cost-effectiveness of services, model the cost and impact of alternative approaches, and identify and address inefficiencies.

There is a growing body of information on the unit cost of evidence-based HIV services in sub-Saharan Africa including HIV testing and counselling (HTC) and the prevention of mother-to-child transmission (PMTCT) [5]. Previous studies have assessed the cost of client-initiated and provider-initiated HTC in facilities [6–17]. A few studies have examined the facility-level cost of PMTCT [7,18–23]. However, with few exceptions [14,15], existing empirical HTC and PMTCT cost data focus on one step of the service cascade – cost per person tested for HTC and cost per woman or per mother–baby pair receiving antiretroviral prophylaxis for PMTCT. These studies do not provide the cost data along service cascades that are critical for identifying and addressing implementation inefficiencies.

In this article, we estimate the average cost per client along the HTC component of the HIV treatment and care cascade [24], which we refer to as the HTC cascade and several steps along the PMTCT cascade [25] across a range of facilities in Kenya, Rwanda, South Africa, and Zambia. We use data from the ‘Optimizing the Response in Prevention: HIV Efficiency in Africa’ (ORPHEA) study [26] – a cross-sectional, micro-costing study conducted from 2012 to 2013 to assess the cost, cost structure, cost variability, and efficiency determinants of HIV prevention interventions.

## Methods

The ORPHEA study methods and tools have been described in detail elsewhere [26], and only those relevant to this analysis are summarized here.

### Study sample

Kenya, Rwanda, South Africa, and Zambia were purposively selected to reflect a range of contextual factors, HIV burden, and HIV prevention intervention coverage levels [26]. For each country, multistage sampling was used to first randomly select subnational areas and then select facilities providing at least one of the following services: HTC, PMTCT, or voluntary medical male circumcision (the latter not reported here). Health facilities in selected subnational areas were enumerated along with information on the services of interest stratified by ownership/management (e.g. owned/managed by government or nongovernment organizations) and level of service provision (e.g. hospitals and primary care clinics). Facilities were randomly sampled within these strata using probability proportional to size, with preference given to integrated sites providing more than one service of interest. A replacement sample was also generated prior to study initiation to systematically substitute facilities in which either services of interest were not provided or service providers declined to participate. Although refusal rates were close to 0 in all countries, replacement sites were included in Kenya when services were found not to be provided in the original sample. The analytic facility samples are shown in Table 1.

### Services studied

*HTC:* The HTC approaches for which costs were estimated were those implemented in sampled facilities at the time of the study: a mix of client-initiated and provider-initiated HTC [27]. The HIV testing algorithms studied were those in place at the time of the study – all countries used serial rapid HIV test algorithms with laboratory ELISA used to resolve discordant results.

*PMTCT:* The following PMTCT components were assessed: HTC, routine clinical monitoring, and provision of antiretroviral and other drugs to mothers and infants. The prophylactic regimens considered were those implemented at the time of the study. In Kenya, South Africa, and Zambia Option A was used, in Rwanda Options B and B+ were implemented (eTable 1) [28,29].

### Ethical clearance

The study was approved by the ethical review boards at the following institutions: National Institute of Public Health, Mexico; Kenyatta National Hospital and University of Nairobi; Northeastern University in Boston; Rwanda Biomedical Center; University of the Witwatersrand in Johannesburg; and University of Zambia [26].

### Data collection

A standardized set of survey instruments were implemented to collect information comparable across countries [26] with data collection staggered by country from October 2012 to December 2013.

Data were collected retrospectively from facility-level and district-level databases, programme records, and reports by month for the calendar year prior to data collection (i.e. 2011 and 2012). Study instruments were designed to assemble information on five cost categories: personnel, recurrent inputs and services, capital (equipment and vehicle operating costs), training, and supervision (staff opportunity costs corresponding to duration of these activities) (eTables 2 and 3, providing information on salaries and input prices). We collected cost data irrespective of funding source, and donated inputs were valued at their opportunity costs determined by local market prices, adopting an economic costing perspective [30].

Time motion [31,32] was implemented at integrated facilities. To assess the contribution of staff for each service in integrated sites, data collectors randomly selected and observed up to six providers per site during different time periods to reflect a representative sample of days, hours, and providers. Data were collected on type and duration of activities, including time spent on administrative work, meetings, and breaks for 3–4 continuous hours. For each multitasking provider observed, time spent with patients for each intervention was assigned to that intervention, and all other noncontact productive time (e.g. administrative work and meetings) was divided equally across the provider's reported service areas. Weights were calculated on the basis of observed staff and applied to all multitasking staff based on provider type and participation across different combinations of services studied. When staff worked on only one service and in facilities where all staff worked on only one service, all effort was allocated to that service.

We collected output data from facility records and from district health information systems – when they were not available at facilities. We gathered information on multiple outputs, corresponding to the following steps along the HTC and PMTCT service cascades, respectively: clients tested and clients diagnosed as HIV-positive; and clients tested, clients diagnosed as HIV-positive, HIV-positive clients receiving antiretroviral prophylaxis, and infants born to HIV-positive clients receiving nevirapine (NVP) (eMethods 1 and 2, providing details on output construction).

### Cost estimation

We estimated costs from the perspective of service providers using a micro-costing approach in which quantity and unit price of essential inputs were assessed along with information on outputs [30]. Total annual costs were calculated for each facility intervention. Costs of personnel, recurrent inputs and services, capital, training, and supervision were aggregated for the year of observation for each intervention as follows: 
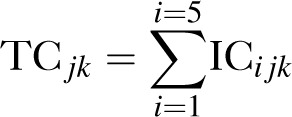


where  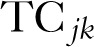 denotes the total annual cost of intervention *j* (1 = HTC and 2 = PMTCT) at facility *k*. The term  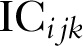 denotes the annual cost of input category *i* (1 = personnel, 2 = recurrent inputs and services, 3 = capital, 4 = training, and 5 = supervision) for intervention *j* at facility *k*.

We estimated the average cost per facility intervention as follows: 
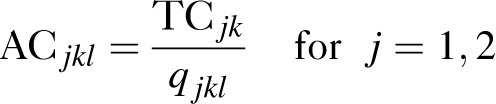


where  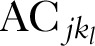 denotes the average cost per output along the cascade for HTC and PMTCT (*j* = 1 or 2);  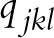 denotes outputs along the cascade where 1=1 for clients tested, 1=2 for clients tested and HIV-positive, 1=3 for HIV-positive clients receiving antiretroviral prophylaxis, and 1=4 for infants born to HIV-positive clients receiving NVP – with the latter two only defined for PMTCT. According to the service cascade definition,  
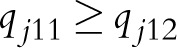
 for HTC and  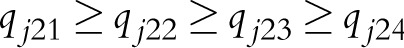 for PMTCT.

For integrated facilities, shared inputs were apportioned across interventions. For recurrent services, capital, training, and supervision, we weighted input annual costs by the annual number of clients per intervention over the total annual number of outpatient clients in the facility. The result was the estimated annual costs allocated to each intervention. Also, as indicated above, to allocate personnel costs across interventions, we applied weights derived from the time motion component.

All cost data were converted from local currencies to United States dollars (US$) using mid-year exchange rates for 2011 (Kenya: 88.81 Kenyan shillings and Zambia: 4860.7 Zambian kwacha) and 2012 (Rwanda: 614.3 Rwandan francs and South Africa: 8.21 South African rand), and then inflated to 2013 prices. We report both unadjusted costs and costs adjusted for purchasing power parity (PPP) for nontradable services, primarily staff salaries.

## Results

### Facility characteristics and average annual outputs

*HTC:* Of the 230 facilities in the HTC sample, 160 were primary care facilities, 67 were secondary hospitals, and three were tertiary sites with over two-thirds managed by government (Table 1). The average annual number of HTC clients per site varied from 1491 (Zambia) to 6610 (Rwanda). HTC clients constituted from 16.2% (Zambia) to 35.4% (Rwanda) of outpatient clients, and the average HTC HIV-positivity rate was 1.6, 11.6, 19.5, and 21.3% in Rwanda, Kenya, Zambia, and South Africa, respectively.

*PMTCT:* Of the 212 facilities in the PMTCT sample, 155 were primary care facilities, 54 were secondary centres, and three were tertiary facilities with over two-thirds managed by government (Table 1). The average annual number of antenatal care (ANC) clients per site ranged from 835 (Rwanda) to 1027 (Zambia). The average PMTCT HIV-positivity rate was 1.7, 10.1, 12.2, and 17.4% in Rwanda, Zambia, Kenya, and South Africa, respectively. PMTCT clients tested for HIV as a proportion of ANC clients ranged from 33.4% (Kenya) to 97.3% (Rwanda). PMTCT clients receiving antiretrovirals for PMTCT as a percentage of ANC clients diagnosed HIV-positive ranged from 73.7% (Zambia) to 91.5% (Kenya). Infants receiving NVP as a proportion of PMTCT clients receiving antiretrovirals for PMTCT ranged from 89.1% (Zambia) to 98.8% (Rwanda).

### Average unit costs along the service cascades

**Fig. 1:**
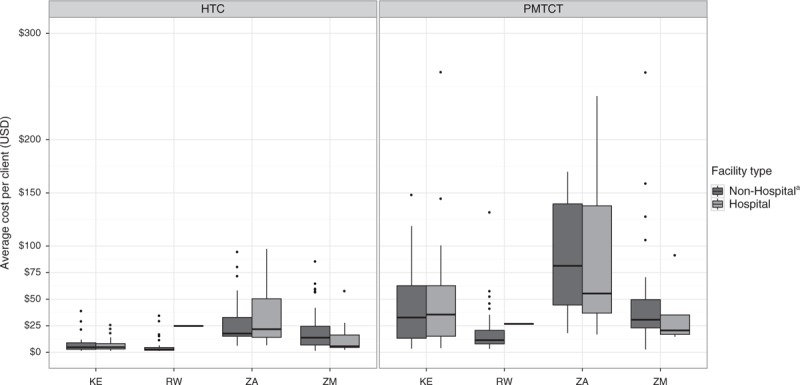
Average cost per client tested for HIV testing and counselling and prevention of mother-to-child transmission by facility type.

HTC, HIV testing and counselling; KE, Kenya; PMTCT, prevention of mother-to-child transmission; RW, Rwanda; SA, South Africa; ZM, Zambia. ^a^’Hospital’ category includes both tertiary and secondary hospitals and ‘non-hospital’ classification includes health centres and other primary care facilities.

*HTC:*Table 2 presents the average unit costs for each cascade step using the full sample of facilities for which unit costs could be estimated. The average cost per HTC client tested ranged from US$5 (SD US$7) in Rwanda to US$31 (SD US$24) in South Africa, whereas the average cost per HTC client diagnosed as HIV-positive ranged from US$122 (SD US$119) in Zambia to US$1367 (SD US$2093) in Rwanda. Table 2 illustrates that variation in these costs also existed within countries. Median unit costs were lower than average unit costs, revealing a skewed distribution of per client costs with some facilities having high costs. This within-country cost variation is further illustrated in Fig. 1, which displays the average cost per HTC client tested by facility type. We did not find differences in the average cost per HTC client tested by facility ownership.

**Fig. 2:**
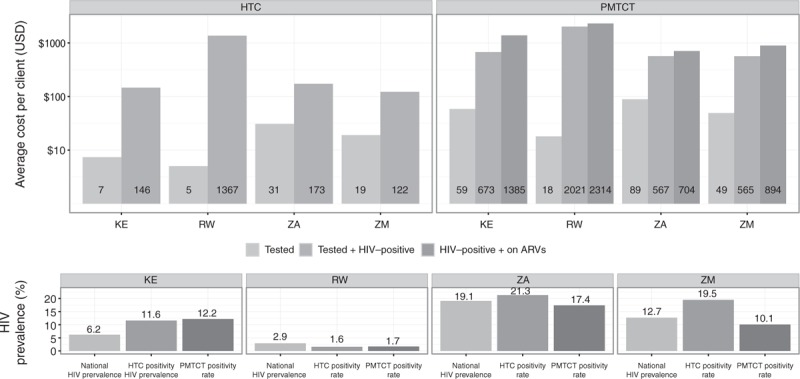
Average cost per client for HIV testing and counselling and prevention of mother-to-child transmission cascades and HIV prevalence.

ARVs, antiretrovirals; HTC, HIV testing and counselling; KE, Kenya; PMTCT, prevention of mother-to-child transmission; RW, Rwanda; SA, South Africa; ZM, Zambia. Labels inside bars indicate precise dollar figures; positivity rate calculated as percentage of (HTC, PMTCT) clients tested and diagnosed HIV-positive as percentage of (HTC, PMTCT) clients tested. National HIV prevalence for the corresponding years retrieved from: World Bank (2014). Data retrieved on December, 2014, from World Development Indicators Online database.

The within country differences between the average cost per HTC client tested and the average cost per HTC client diagnosed as HIV-positive were associated with the positivity rates in the HTC samples (Fig. 2). The difference was the largest in Rwanda where the HIV-positivity rate was 1.6%, almost half the national prevalence.

As regards between country differences, after adjusting for differences in prices using PPP-adjusted dollars, the average cost per HTC client tested in Rwanda and Kenya was significantly lower than in South Africa (*P* < 0.01) and Zambia (*P* < 0.01), and the average cost per HTC client diagnosed as HIV-positive was significantly higher in Rwanda than in the other three countries (*P* < 0.01).

*PMTCT:* The average annual cost per PMTCT client tested ranged from US$18 (SD US$20) in Rwanda to US$89 (SD US$56) in South Africa (Table 2). The average cost per PMTCT client diagnosed as HIV-positive ranged from US$565 (SD US$584) in Zambia to US$2021 (SD US$3210) in Rwanda. The average cost per PMTCT client receiving antiretrovirals ranged from US$704 (SD US$610) in South Africa to US$2314 (SD US$3204) in Rwanda. The average cost per infant receiving NVP ranged from US$888 (SD US$884) in South Africa to US$2359 (SD US$3257) in Rwanda. Note that these average annual costs were estimated using different sample sizes along the PMTCT service cascade due to missing values for some output indicators (eTable 4, providing the average unit costs for the subset of facilities for which unit costs could be estimated at every step of the PMTCT cascade). As with the costs along the HTC cascade, Table 2 displays considerable variation within countries in the average costs along the PMTCT service cascade. This within-country cost variation is further illustrated in Fig. 1, which displays the average cost per PMTCT client tested by facility type. We did not find differences in the average cost per PMTCT client tested by facility ownership.

As with the costs along the HTC cascade, the differences between the average cost per PMTCT client tested and the average cost per PMTCT client diagnosed as HIV-positive were closely associated with the PMTCT positivity rate and the largest in Rwanda (Fig. 2). In contrast, the difference between the average cost per PMTCT client tested and diagnosed as HIV-positive and the average cost per client receiving antiretroviral prophylaxis was the smallest in Rwanda and the largest in Kenya.

In terms of the between-country differences, when adjusting for differences in prices, the average cost per PMTCT client tested in Rwanda was significantly lower than in Kenya (*P* < 0.01), South Africa (*P* < 0.01), and Zambia (*P* < 0.05), and the average cost per PMTCT client diagnosed as HIV-positive was significantly higher in Rwanda than in South Africa (*P* < 0.01) and Zambia (*P* < 0.05).

### Distribution of costs

**Fig. 3:**
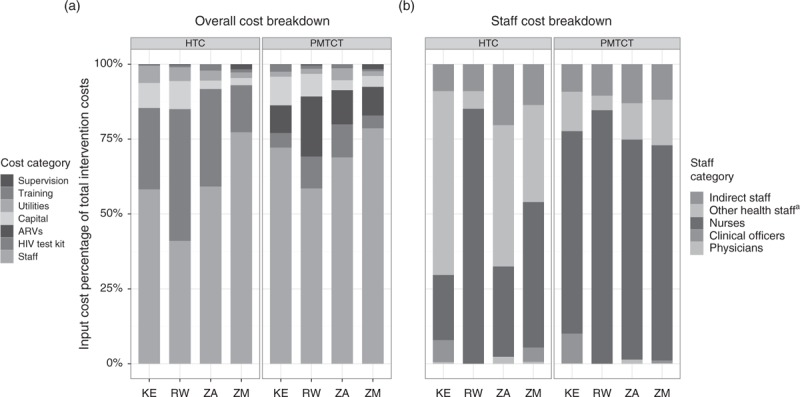
Breakdown of inputs and staff composition for HIV testing and counselling and prevention of mother-to-child transmission.

ARVs, antiretrovirals; HTC, HIV testing and counselling; KE, Kenya; PMTCT, prevention of mother-to-child transmission; RW, Rwanda; SA, South Africa; ZM, Zambia. (a) Overall cost breakdown and (b) staff cost breakdown. ^a^Other health staff include counsellors, social workers, community health workers, peer educators, laboratory technicians, pharmacists, and volunteers.

Staff costs comprised the majority of costs for both interventions in Kenya, South Africa, and Zambia (Fig. 3). Supplies – with HIV test kits as the main contributor – made up the second largest shares of HTC costs in these countries, and drugs made up the second largest proportion of PMTCT costs. In Rwanda, although the pattern for PMTCT was similar to that in the other countries, for HTC, staff costs made up a smaller proportion of total HTC costs, and the proportions of staff and supply costs were almost equivalent.

Figure 3 also shows the average number of full-time equivalent staff for each intervention in the four countries. Other health staff, comprising mostly of counsellors, represented an important HTC provider category for Kenya, South Africa, and Zambia, whereas nurses dominated the provision of HTC in Rwanda. In contrast, the cost of PMTCT service provision was dominated by nursing costs in all countries.

### Discussion

The current cost analysis of HTC and PMTCT services in Kenya, Rwanda, South Africa, and Zambia is unique in its focus on estimating costs along the service cascades of these two interventions. It also represents one of the most comprehensive, multicountry assessments of HTC and PMTCT service delivery costs. The sample size was large compared with similar studies, and data were collected using robust standardized methods and tools yielding comparable information across facilities within and across countries.

We found between-country differences in the first and second steps of the HTC and PMTCT cascades; in general, after adjusting for differences in prices, the average cost per client tested was lower in Rwanda, whereas the average cost per client diagnosed as HIV-positive was higher for both HTC and PMTCT. We also observed substantial variation in the unit costs of HTC and PMTCT within facility types in South Africa, Kenya, and Zambia and to a lesser extent among primary care facilities in Rwanda (only one hospital included in the sample) suggesting the existence of implementation inefficiencies. Staff costs were the most important driver of HTC and PMTCT service delivery costs in all countries except Rwanda where staff costs made up the majority of PMTCT costs but less than half of HTC costs. Cross-country salary differences – even after controlling for purchasing power – were a determinant of the cross-country unit cost variation; average salaries for all provider types were significantly higher in South Africa, whereas salaries for nurses were significantly lower in Rwanda, for example. Also relevant to the cross-country differences in unit costs and cost compositions were the variations in the services delivered and the staff categories delivering services. Nurses dominating HTC provision in Rwanda in contrast to counsellors dominating HTC provision in the other countries is likely explained by the prevalence of provider-initiated testing in Rwanda at the time of the study compared with the pervasiveness of client-initiated HTC in the other countries. Similarly, for PMTCT, the larger proportion of antiretroviral costs in Rwanda was due to the fact that Rwanda was the only country implementing Options B and B+ at the time of data collection.

We found unit cost heterogeneity along the HTC and PMTCT cascades within countries. Specifically, there were large differences between the unit costs of the first and second steps of the HTC cascade within each country, although the difference was the largest in Rwanda. Although average cost per person tested was the lowest in Rwanda, average cost per person testing positive was the highest. This difference was driven by the low positivity rate in the Rwandan sample; in Rwanda, many more people were tested to diagnose one person living with HIV. This finding suggests that efficiency gains could be made by better targeting HTC services to populations at risk for HIV infection. Unfortunately, we did not collect data on whether positive tests were first-time tests or repeat tests and therefore cannot determine the cost of identifying new HIV cases. Given the high levels of coverage of HTC and antiretroviral treatment achieved in Rwanda [33], the high cost per person diagnosed may also reflect the increasing marginal cost of finding new HIV cases as HTC coverage expands.

For PMTCT, we found differences between the unit costs of the first and second steps, and the difference was the largest in Rwanda. We also observed large differences between the unit costs of the second and third steps, which provide insights into the linkages of HIV-positive women to prophylaxis and care and are underpinned by retention along the cascade within given facilities. The difference between the second and third steps was greater in Kenya, where less than half the women identified as positive received antiretroviral prophylaxis in the facilities where they were tested; followed by women in Zambia, where the equivalent figure was roughly three quarters. Though we were unable to identify whether women received antiretroviral drugs in another facility as previous studies have done [34–38], our findings raise concerns about potentially large losses along the PMTCT cascade most markedly in Kenya and Zambia.

When compared with prior studies that assessed both, the average costs per HTC client tested in our study were similar to those found in prior studies, whereas the average costs per client diagnosed HIV-positive were somewhat higher [14,15]; we are not aware of similar studies conducted for PMTCT. In a previous study that collected data for 2008–2009 in Kenya and Swaziland, unit cost per client tested was between US$6 and 9, whereas unit cost per client diagnosed as HIV-positive ranged from US$47 to 110 [15]. In a study that collected data from 2003 to 2005 in Uganda, facility-based HCT costs ranged between US$12 and 19 and US$43 and 101 per client tested and client diagnosed HIV-positive, respectively. Most of the distinctions are probably due to differences in facility-level positivity rates. Other likely contributing factors are variations in health systems set-up and distinctions in indicator definitions and methods used. Our study is the only one that used time motion direct observation methods to assess staff time use, the dominant cost component of these interventions.

The following limitations should be considered when interpreting our findings. Though our study is unique in its focus on multiple steps along the HTC and PMTCT service cascades, there are some shortcomings. The study did not capture data on the critical step following HIV diagnosis: linkage and enrolment in care [24]. We were also unable to capture details on monitoring and quality assurance. Though systematic sampling was used, the selected samples are unlikely to be nationally representative as subnational areas were purposefully selected to represent in-country variation in HIV epidemics. Because we retrospectively collected information on inputs, costs, and outputs, staff observations were made subsequent to the period studied, although never more than 12 months later. We used routine monitoring data to capture information on outputs, and the detail, quality, and completeness of these data varied. Our inability to distinguish between client-initiated and provider-initiated HTC is one drawback of the information used. Every effort was made to capture staff costs as accurately as possible through time-motion methods that sought to be as inconspicuous as feasible; however, there are limitations to these methods including the potential bias introduced by the observer effect as well as the possibility that the days and hours of staff moments monitored were not representative of the entirety of staff moments in a given year [32].

In conclusion, we found important differences in unit costs along the HTC and PMTCT service cascades within and between countries. Although variation in service quality might explain some of this heterogeneity, it is unlikely that the magnitude of differences observed can be explained by differences in service quality alone. Rather, this cost variation suggests that substantial efficiency gains could be made in delivering these services in all countries studied whether by better targeting services, altering who delivers services, or changing the way services are delivered. To provide guidance to policymakers, we are undertaking further work to assess the determinants of this cost variation.

## Acknowledgements

S.B.A. and S.G.S.R. conceived the overall study and oversaw study design, data collection, data analysis, interpretation of data, and preparation of manuscript. M.O. drafted the manuscript. D.C.L. led data analysis with contributions from I.O.M. A.K. designed and led the implementation of the time-motion component. C.C. supported the overall implementation of the study. O.G., R.W., and J.W. (Kenya); J.C. and S.N. (Rwanda); A.C. and F.M. (Zambia); N.M. (South Africa) led data collection in the countries noted. All authors contributed to the design of the study. All authors participated in data interpretation, contributed to subsequent drafts of the manuscript, and read and approved the final manuscript.

Additional members of the ORPHEA study team: *Mexico*: Amilcar Azamar-Alonso and Gina La Hera Fuentes of the National Institute of Public Health, Center for Health Systems Research, Cuernavaca, Mexico; Martin Romero-Martínez of the National Institute of Public Health, Division of Surveys, Cuernavaca, Mexico; Raluca Buzdugan of University of California, Berkeley, School of Public Health, California, USA; Alvarao Canales and Victor Canales of Sistemas Integrales, Santiago, Chile; and Rita Cuckovich of Consorcio de Investigación sobre VIH/SIDA/TB, Cuernavaca, Mexico. *Kenya*: Mercy Mugo and Hellen Nyakundi of University of Nairobi, School of Economics and School of Public Health, Nairobi, Kenya. *Rwanda*: Jean Pierre Ayingoma, Placidie Mugwaneza, and Eric Remera of Rwanda Biomedical Center, Institute of HIV/AIDS, Disease Prevention & Control, Kigali, Rwanda; and Collins Kamanzi, Nathalie Mulindahabi, Angele Musabyimana, and Sabine Musange of National University of Rwanda School of Public Health, Kigali, Rwanda. *South Africa*: Jenny Coetzee, Charity Dire, Limakatso Lebina, and Sabelo Sekhukuni of Perinatal HIV Research Unit, University of the Witwatersrand, Johannesburg, South Africa. *Zambia*: Sydney Chauwa and Bona Chitah of University of Zambia, Division of Economics, Lusaka, Zambia; Kumbutso Dzekedzeke, Dzekdzeke Research & Consultancy, Lusaka, Zambia.

Funding source: This study was conducted with funding from the Bill and Melinda Gates Foundation.

Disclaimer: The findings and conclusions in this article are those of the authors and do not necessarily represent the positions of the institutions with which they are affiliated. The views expressed by Marjorie Opuni are her own and do not reflect the official position of UNAIDS.

### Conflicts of interest

There are no conflicts of interest.

## Supplementary Material

Supplemental Digital Content

## Figures and Tables

**Table 1:** Sample sites by ownership and facility type and key cascade indicators.

Countries	Kenya		Rwanda		South Africa		Zambia	
Subnational areas	10 out of 47 counties (purposively)		All five provinces; 26 out of 30 districts (randomly)		Three out of nine provinces (purposively); six out of 52 districts (randomly)		Five out of nine provinces (purposively); 21 out of 72 districts (randomly)	
Random sample of sites	HTC	PMTCT	HTC	PMTCT	HTC	PMTCT	HTC	PMTCT
Tertiary hospitals	1	2			2	1		
Secondary hospitals	30	31	1	1	23	10	13	12
Health centres	15	17	52	52	13	13	42	42
Other primary care facilities^a^	10	7			23	20	5	4
Total	56	57	53	53	61	44	60	58
Ownership								
Public	29	37	29	29	52	36	52	51
Private^b^	27	20	24	24	9	8	8	7
Cascade indicators								
Average annual number of clients tested	4235	864	6610	835	4784	989	1491	1027
Average % of total patients tested^c^	30.2	33.4	35.4	97.3	16.9	43.1	16.2	60.2
Average % of clients diagnosed HIV-positive	11.6	12.2	1.6	1.7	21.3	17.4	19.5	10.1
Average % of clients receiving ARV prophylaxis	NA	91.5	NA	86.3	NA	83.3	NA	73.7
Average % of infants receiving NVP prophylaxis	NA	96.1	NA	98.8	NA	98.0	NA	89.1

ARV, antiretroviral; HTC, HIV testing and counselling; NVP, nevirapine; PMTCT, prevention of mother-to-child transmission.

^a^Other primary care facilities include the following facilities: in Kenya – dispensaries, medical clinics, and mobile clinics; in South Africa – medical clinics; and in Zambia – health posts.

^b^Private facilities are not-for-profit institutions operated by faith-based and other nongovernmental organizations.

^c^For HTC, the denominator is the total annual number of outpatient clients; for PMTCT the denominator is the total annual number of antenatal care clients.

**Table 2:** Average annual unit costs along the HIV testing and counselling and prevention of mother-to-child transmission service cascades (United States dollars).

	Kenya					Rwanda					South Africa					Zambia				
	*n*	Mean	SD	Median	Wt mean^a^	*n*	Mean	SD	Median	Wt mean^a^	*n*	Mean	SD	Median	Wt mean^a^	*n*	Mean	SD	Median	Wt mean^a^
HTC																				
Average cost per HTC client tested	56	7	7	5	7	53	5	7	3	5	61	31	24	21	31	60	19	19	13	13
Average cost per HTC client diagnosed HIV-positive	56	146	318	55	80	53	1367	2093	591	1057	59	173	157	126	177	58	122	119	90	85
Average cost per HTC client tested (PPP)^b^	56	15	16	9	12	53	9	13	4	8	61	43	36	26	43	60	35	35	24	23
Average cost per HTC client diagnosed HIV-positive (PPP)	56	291	692	102	146	53	1976	2679	735	1558	59	243	240	161	250	58	222	225	164	153
PMTCT																				
Average cost per PMTCT client tested	57	59	84	35	49	53	18	20	11	16	44	89	56	81	99	58	49	71	30	35
Average cost per PMTCT client diagnosed HIV-positive	51	673	1037	256	776	53	2021	3210	1159	1582	42	567	417	469	509	56	565	584	402	360
Average cost per PMTCT client receiving ARV prophylaxis	31	1385	3750	275	1262	53	2314	3204	1393	1868	34	704	610	528	765	39	894	1117	483	535
Average cost per infant receiving NVP prophylaxis	26	1329	3965	304	1112	51	2359	3257	1393	1887	10	888	884	462	1085	32	1072	1229	529	650
Average cost per PMTCT client tested (PPP)	57	130	197	71	106	53	34	42	19	29	43	125	81	118	146	58	89	132	55	61
Average cost per PMTCT client diagnosed HIV-positive (PPP)	51	1456	2323	544	1671	53	3943	6807	1830	3042	41	803	627	688	749	56	1025	1105	683	640
Average cost per PMTCT client receiving ARV prophylaxis (PPP)	31	3041	8587	540	2735	53	4496	6806	2546	3568	34	1031	942	757	1120	39	1618	2112	844	944
Average cost per infant receiving NVP prophylaxis (PPP)	26	2921	9109	605	2404	51	4581	6922	2546	3600	10	1290	1364	607	1599	32	1936	2325	925	1125

ARV, antiretroviral; HTC, HIV testing and counselling; *n*, sample size; NVP, nevirapine; PMTCT, prevention of mother-to-child transmission; PPP, purchasing power parity; Wt mean, weighted mean; Unit costs in 2013 US$.

^a^Weighted mean represents a nationally representative average value, taking into account the relative contribution of each facility in terms of its patient volume. It was calculated as the summation of each data point multiplied by a nonnegative weight (defined as the number of annual HTC clients/outpatient health clients). Therefore, data points with a higher weight contribute more to the weighted mean than do elements with a low weight.

^b^Staff costs (nontradable inputs) were adjusted for purchasing power parity differences between countries using 2013 World Bank Data (*source:* http://data.worldbank.org/indicator/PA.NUS.PPP).

## References

[1] UNAIDS. Fast track: ending the AIDS epidemic by 2030. Geneva: UNAIDS; 2014.

[2] UNAIDS. Together we will end AIDS. Geneva: Joint United Nations Programme on HIV/AIDS; 2012.

[3] Schwartlander B Stover J Hallett T Atun R Avila C Gouws E Towards an improved investment approach for an effective response to HIV/AIDS. *Lancet* 2011; 377:2031–2041. 21641026 10.1016/S0140-6736(11)60702-2

[4] Vassall A Remme M Watts C Hallett T Siapka M Vickerman P Financing essential HIV services: a new economic agenda. *PLoS Med* 2013; 10:e1001567. 24358028 10.1371/journal.pmed.1001567 PMC3866083

[5] Siapka M Remme M Obure CD Maier CB Dehne KL Vassall A Is there scope for cost savings and efficiency gains in HIV services? A systematic review of the evidence from low- and middle-income countries. *Bull World Health Organ* 2014; 92:499AD–511AD. 25110375 10.2471/BLT.13.127639 PMC4121865

[6] Aliyu HB Chuku NN Kola-Jebutu A Abubakar Z Torpey K Chabikuli ON What is the cost of providing outpatient HIV counseling and testing and antiretroviral therapy services in selected public health facilities in Nigeria?. *J Acquir Immune Defic Syndr* 2012; 61:221–225. 22820805 10.1097/QAI.0b013e3182683b04

[7] Bratt JH Torpey K Kabaso M Gondwe Y Costs of HIV/AIDS outpatient services delivered through Zambian public health facilities. *Trop Med Int Health* 2011; 16:110–118. 20958891 10.1111/j.1365-3156.2010.02640.x

[8] Forsythe S Arthur G Ngatia G Mutemi R Odhiambo J Gilks C Assessing the cost and willingness to pay for voluntary HIV counselling and testing in Kenya. *Health Policy Plan* 2002; 17:187–195. 12000779 10.1093/heapol/17.2.187

[9] Grabbe KL Menzies N Taegtmeyer M Emukule G Angala P Mwega I Increasing access to HIV counseling and testing through mobile services in Kenya: strategies, utilization, and cost-effectiveness. *J Acquir Immune Defic Syndr* 2010; 54:317–323. 20453819 10.1097/QAI.0b013e3181ced126 PMC3225204

[10] Hausler HP Sinanovic E Kumaranayake L Naidoo P Schoeman H Karpakis B Costs of measures to control tuberculosis/HIV in public primary care facilities in Cape Town, South Africa. *Bull World Health Organ* 2006; 84:528–536. 16878226 10.2471/blt.04.018606 PMC2627402

[11] John FN Farquhar C Kiarie JN Kabura MN John-Stewart GC Cost effectiveness of couple counselling to enhance infant HIV-1 prevention. *Int J STD AIDS* 2008; 19:406–409. 18595879 10.1258/ijsa.2008.007234 PMC2765914

[12] Marseille E Dandona L Saba J McConnel C Rollins B Gaist P Assessing the efficiency of HIV prevention around the world: methods of the PANCEA project. *Health Serv Res* 2004; 39:1993–2012. 15544641 10.1111/j.1475-6773.2004.00329.x PMC1361109

[13] McConnel CE Stanley N du Plessis JA Pitter CS Abdulla F Coovadia HM The cost of a rapid-test VCT clinic in South Africa. *S Afr Med J* 2005; 95:968–971. 16465359

[14] Menzies N Abang B Wanyenze R Nuwaha F Mugisha B Coutinho A The costs and effectiveness of four HIV counseling and testing strategies in Uganda. *AIDS* 2009; 23:395–401. 19114865 10.1097/QAD.0b013e328321e40b

[15] Obure CD Vassall A Michaels C Terris-Prestholt F Mayhew S Stackpool-Moore L Optimising the cost and delivery of HIV counselling and testing services in Kenya and Swaziland. *Sex Transm Infect* 2012; 88:498–503. 22859498 10.1136/sextrans-2012-050544 PMC3595498

[16] Sweat M Gregorich S Sangiwa G Furlonge C Balmer D Kamenga C Cost-effectiveness of voluntary HIV-1 counselling and testing in reducing sexual transmission of HIV-1 in Kenya and Tanzania. *Lancet* 2000; 356:113–121. 10963247 10.1016/S0140-6736(00)02447-8

[17] Terris-Prestholt F Kumaranayake L Foster S Kamali A Kinsman J Basajja V The role of community acceptance over time for costs of HIV and STI prevention interventions: analysis of the Masaka Intervention Trial, Uganda, 1996–1999. *Sex Transm Dis* 2006; 33:S111–116. 16505738 10.1097/01.olq.0000175389.10289.ba

[18] Orlando S Marazzi MC Mancinelli S Liotta G Ceffa S Giglio P Cost-effectiveness of using HAART in prevention of mother-to-child transmission in the DREAM-Project Malawi. *J Acquir Immune Defic Syndr* 2010; 55:631–634. 21934555 10.1097/QAI.0b013e3181f9f9f5

[19] Robberstad B Evjen-Olsen B Preventing mother to child transmission of HIV with highly active antiretroviral treatment in Tanzania – a prospective cost-effectiveness study. *J Acquir Immune Defic Syndr* 2010; 55:397–403. 20739897 10.1097/QAI.0b013e3181eef4d3

[20] Scott CA Iyer HS Lembela Bwalya D Bweupe M Rosen SB Scott N Uptake, outcomes, and costs of antenatal, well baby, and prevention of mother-to-child transmission of HIV services under routine care conditions in Zambia. *PLoS One* 2013; 8:e72444. 24015245 10.1371/journal.pone.0072444 PMC3756060

[21] Stringer EM Sinkala M Stringer JS Mzyece E Makuka I Goldenberg RL Prevention of mother-to-child transmission of HIV in Africa: successes and challenges in scaling-up a nevirapine-based program in Lusaka, Zambia. *AIDS* 2003; 17:1377–1382. 12799559 10.1097/01.aids.0000060395.18106.80 PMC2745990

[22] Toure H Audibert M Doughty P Tsague L Mugwaneza P Nyankesha E Public sector services for the prevention of mother-to-child transmission of HIV infection: a micro-costing survey in Namibia and Rwanda. *Bull World Health Organ* 2013; 91:407–415. 24052677 10.2471/BLT.12.113639 PMC3777138

[23] Zulliger R Black S Holtgrave DR Ciaranello AL Bekker LG Myer L Cost-effectiveness of a package of interventions for expedited antiretroviral therapy initiation during pregnancy in Cape Town, South Africa. *AIDS Behav* 2014; 18:697–705. 24122044 10.1007/s10461-013-0641-7 PMC3984926

[24] WHO. Consolidated guidelines on the use of antiretroviral drugs for treating and preventing HIV infection: recommendations for a public health approach June 2013. Geneva: WHO; 2013. 24716260

[25] UNICEF. Consultative meeting on evaluating the impact of prevention of mother-to-child transmission of HIV (PMTCT) services in low- and middle-income countries in averting new HIV infections in children and improving child survival. New York City: UNICEF; 2009.

[26] Bautista-Arredondo S Sosa-Rubi SG Opuni M Kwan A Chaumont C Coetzee J Assessing cost and technical efficiency of HIV prevention interventions in sub-Saharan Africa: the ORPHEA study design and methods. *BMC Health Serv Res* 2014; 14:599. 25927555 10.1186/s12913-014-0599-9 PMC4260235

[27] WHO, UNAIDS. Guidance on provider-initiated HIV testing and counselling in health facilities. Geneva: WHO; 2007.

[28] WHO. Antiretroviral drugs for treating pregnant women and preventing HIV infection in infants: Recommendations for a public health approach. Geneva: WHO; 2010. 26180894

[29] WHO. Use of antiretroviral drugs for treating pregnant women and preventing HIV infection in infants: programmatic update. Geneva: WHO; 2012.

[30] Drummond MF Sculpher MJ Torrence DW O’Brien BJ Stoddart GL Methods for the economic evaluation of healthcare programmes. 3rd ed. Oxford: Oxford University Press; 2005.

[31] Adam T Sources of variability in costing methods: implications for transferability of cost-effectiveness results. Rotterdam: Erasmus University; 2006.

[32] Bratt JH Foreit J Chen PL West C Janowitz B de Vargas T A comparison of four approaches for measuring clinician time use. *Health Policy Plan* 1999; 14:374–381. 10787653 10.1093/heapol/14.4.374

[33] UNAIDS. The gap report. Geneva: UNAIDS; 2014.

[34] Barker PM Mphatswe W Rollins N Antiretroviral drugs in the cupboard are not enough: the impact of health systems’ performance on mother-to-child transmission of HIV. *J Acquir Immune Defic Syndr* 2011; 56:e45–e48. 21084998 10.1097/QAI.0b013e3181fdbf20

[35] Ferguson L Lewis J Grant AD Watson-Jones D Vusha S Ong’ech JO Patient attrition between diagnosis with HIV in pregnancy-related services and long-term HIV care and treatment services in Kenya: a retrospective study. *J Acquir Immune Defic Syndr* 2012; 60:e90–e97. 22421747 10.1097/QAI.0b013e318253258a

[36] Stringer EM Ekouevi DK Coetzee D Tih PM Creek TL Stinson K Coverage of nevirapine-based services to prevent mother-to-child HIV transmission in 4 African countries. *JAMA* 2010; 304:293–302. 20639563 10.1001/jama.2010.990

[37] Turan JM Bukusi EA Onono M Holzemer WL Miller S Cohen CR HIV/AIDS stigma and refusal of HIV testing among pregnant women in rural Kenya: results from the MAMAS study. *AIDS Behav* 2011; 15:1111–1120. 20827573 10.1007/s10461-010-9798-5 PMC3127002

[38] Turan JM Nyblade L HIV-related stigma as a barrier to achievement of global PMTCT and maternal health goals: a review of the evidence. *AIDS Behav* 2013; 17:2528–2539. 23474643 10.1007/s10461-013-0446-8

