# Supplementary material for: Costs along the service cascades for HIV testing and counselling and prevention of mother-to-child transmission
Source: AIDS. 2016 Sep 28;30(16):2495–504. doi: 10.1097/QAD.0000000000001208 (PMC5051528; doi:10.1097/QAD.0000000000001208)
Supplement: Supplemental Digital Content [file aids-30-2495-s001.docx]

**Supplementary Online Content**

**eTable 1: PMTCT prophylaxis regimens costed**

|  | **Kenya** | **Rwanda** | **South Africa** | **Zambia** |
| --- | --- | --- | --- | --- |
| Regimen | Option A | Option B/ B+ | Option A | Option A |
| During pregnancy^a^ | AZT | TDF/3TC/EFV | AZT | AZT |
| Post delivery |  |  |  |  |
| Mothers | AZT/3TC (7 days) | TDF/3TC/EFV | sd TDF/FTC | AZT/3TC (7 days) |
| Infants^b^ | NVP | NVP | NVP | NVP |
|  | CTX | CTX | CTX | CTX |

AZT, zidovudine; 3TC, lamivudine; EFV, efavirenz; FTC, Emcitricitabine; TDF, Tenofovir; sd, single dose; NVP, nevirapine; CTX, Cotrimoxazole;

^a^6 months of AZT costed for Kenya, South Africa, and Zambia and 12 months of TDF/3TC/EFV assumed for Rwanda; ^b^6 months of NVP and Cotrimoxazole costed for all countries

**eTable 2: Staff salaries (US$)**

|  | | **Kenya** | | | | | | | | **Rwanda** | | | | | | | | **South Africa** | | | | | | | | **Zambia** | | | | | | | |
| --- | --- | --- | --- | --- | --- | --- | --- | --- | --- | --- | --- | --- | --- | --- | --- | --- | --- | --- | --- | --- | --- | --- | --- | --- | --- | --- | --- | --- | --- | --- | --- | --- | --- |
|  | | n^a^ | | Median | | Mean | | SD | | n^a^ | | Median | | Mean | | SD | | n^a^ | | Median | | Mean | | SD | | n^a^ | | Median | | Mean | | SD | |
| Average annual salary of physicians | | 11 | | 26,241 | | 21,519 | | 11,749 | | 11 | | 19,312 | | 21,533 | | 3,175 | | 61 | | 53,634 | | 68,800 | | 62,276 | | 8 | | 29,735 | | 28,483 | | 9,706 | |
| Average annual salary of clinical officers | | 102 | | 16,922 | | 16,365 | | 11,490 | | - | | - | | - | | - | | - | | - | | - | | - | | 41 | | 16,115 | | 16,020 | | 1,628 | |
| Average annual salary of nurses | | 405 | | 10,202 | | 11,311 | | 8,208 | | 574 | | 6,259 | | 7,560 | | 6,815 | | 978 | | 33,976 | | 45,569 | | 31,616 | | 424 | | 15,736 | | 16,022 | | 2,585 | |
| Average annual salary of other health staff^b^ | | 433 | | 8,257 | | 9,338 | | 7,477 | | 89 | | 6,259 | | 6,747 | | 4,625 | | 387 | | 26,756 | | 56,395 | | 55,034 | | 384 | | 2,270 | | 5,151 | | 5,841 | |
| Average annual salary of indirect staff^c^ | | 233 | | 5,248 | | 7,855 | | 9,285 | | 143 | | 5,736 | | 6,247 | | 5,790 | | 387 | | 28,117 | | 28,289 | | 24,954 | | 114 | | 5,621 | | 6,632 | | 3,610 | |

n, sample size; SD, standard deviation; All salaries in 2013 US Dollars

^a^Sample sizes for staff salaries denote the number of staff assessed

^b^Other health staff include: counselors, social workers, community health workers, peer educators, laboratory technicians, pharmacists, and volunteers

^c^Indirect staff includes management, clerical, cleaning, maintenance and security providers

**eTable 3: Prices of HIV test kits and drug regimens**

|  | Kenya | Rwanda | South Africa | Zambia |
| --- | --- | --- | --- | --- |
| **RAPID HIV TEST KITS**^a^ |  |  |  |  |
| Rapid HIV test kit (Determine) | 0.85 | 1.10 | 4.65 | 0.70 |
| Rapid HIV test kit (Unigold ) | 1.18 | 1.90 | - | 1.78 |
| **ARVS AND PROPHYLAXIS** |  |  |  |  |
| Option B/B^b^ | - | 141.12 | - | - |
| Option A^c^ | 42.94 |  | 46.40 | 45.93 |
| NVP infant prophylaxis^d^ | 2.19 | 0.32 | 3.60 | 4.23 |
| Infant CTX prophylaxis^e^ | 2.25 | 2.25 | 2.25 | 2.25 |

NVP, Nevirapine; CTX, Cotrimoxazole

^a^Only one price for HIV test kits in South Africa

^b^Option B/B+ in Rwanda includes 12 months of TDF/3TC/EFV, where TDF, Tenofovir; 3TC, Lamivudine; EFV, Efavirenz

^c^Option A includes for Kenya and Zambia: AZT 6 months, AZT+3TC 7 days; for South Africa: AZT 6 months, sd-TDF+FTC, where AZT, Zidovudine; 3TC, Lamivudine; FTC, Emcitricitabine; TDF, Tenofovir

^d^6 months NVP infant prophylaxis

^e^Price of infant co-trimoxazole in Rwanda; 6 months infant CTX prophylaxis

**eMethods 1: Assumptions on HTC outputs**

The following questions were asked at facility-level to collect information about HTC outputs along the service cascade:

- 1. What was the number of HIV tests administered in TOTAL in last calendar year?
  2. What was the number of HIV tests administered in MONTH/last calendar year?
  3. What was the number of individuals attending HTC in TOTAL in last calendar year?
  4. What was the number of individuals attending HTC in MONTH/last calendar year?

In most cases, the question that was used to inform the output indicator was the one on the total annual number of HIV tests administered (question 1). Whenever this information was missing, we used the arithmetic sum of the monthly information provided by question 2. In those cases where answers were missing for both questions 1 and 2, we used the information provided on the individuals attending HTC (questions 3 or 4). When the information on a given month was missing, we imputed the mean monthly output for the months completed.

**eMethods 2: Assumptions on PMTCT outputs**

The following PMTCT outputs along service cascade were used:

- PMTCT clients (Number of pregnant women attending antenatal care (old+new visits)),
- PMTCT clients tested (Antenatal clients tested at first antenatal visit + Antenatal clients tested at follow-up antenatal visits),
- PMTCT clients diagnosed HIV-positive,
- PMTCT clients receiving ARV prophylaxis (according to the prophylaxis regime of each country) and
- PMTCT infants receiving prophylaxis (prophylaxis for infants during the first 6 weeks)

We used the following questions to calculate the number of PMTCT clients:

- What was the number of this (output indicator) in TOTAL in last calendar year?
- What was the number of this (output indicator) in (MONTH)/last calendar year?

Whenever the records showed more clients in a consecutive step in the service cascade, the later indicator was censored with the figure of the previous step, e.g. if there were more women on ARV prophylaxis than HIV-positive women, we assumed the number of women on ARV prophylaxis to be equal to the number of HIV-positive women. Additionally, backward imputation was performed when we had a single missing value in the service cascade continuum. For example, when there was no record of the number of women on ARV prophylaxis but the number of infants on prophylaxis was available, we set the number of women on ARV prophylaxis to be equal to the number of infants on prophylaxis.

**eTable 4: Average annual unit costs along the PMTCT service cascades for a subsample of facilities with complete indicators of the service cascade (US$)**

|  | Kenya | | | | | Rwanda | | | | | South Africa | | | | | Zambia | | | | |
| --- | --- | --- | --- | --- | --- | --- | --- | --- | --- | --- | --- | --- | --- | --- | --- | --- | --- | --- | --- | --- |
|  | N | Mean | SD | Median | Wt mean^a^ | N | Mean | SD | Median | Wt mean^a^ | N | Mean | SD | Median | Wt mean^a^ | N | Mean | SD | Median | Wt mean^a^ |
| **PMTCT** |  |  |  |  |  |  |  |  |  |  |  |  |  |  |  |  |  |  |  |  |
| Average cost per PMTCT client tested | 26 | 29 | 23 | 22 | 26 | 51 | 16 | 12 | 11 | 14 | 10 | 75 | 59 | 47 | 97 | 32 | 51 | 85 | 29 | 31 |
| Average cost per PMTCT client diagnosed HIV-positive | 26 | 616 | 864 | 232 | 761 | 51 | 2,048 | 3,270 | 1,159 | 1,586 | 10 | 616 | 561 | 395 | 739 | 32 | 531 | 658 | 345 | 292 |
| Average cost per PMTCT client receiving ARV prophylaxis | 26 | 1,304 | 3,970 | 274 | 1,207 | 51 | 2,352 | 3,261 | 1,393 | 1,879 | 10 | 883 | 889 | 434 | 1,078 | 32 | 958 | 1,212 | 471 | 525 |
| Average cost per infant receiving NVP prophylaxis | 26 | 1,329 | 3,965 | 304 | 1,246 | 51 | 2,359 | 3,257 | 1,393 | 1,887 | 10 | 888 | 884 | 462 | 1,085 | 32 | 1,072 | 1,229 | 529 | 653 |

The unit costs described in this table refer to the sample of facilities for which the unit costs could be estimated at every step of the service cascade, i.e. this subsample is defined by the number of facilities with an available figure for the cost per infant receiving NVP prophylaxis.

N, sample size; SD, standard deviation; Wt mean, weighted mean; HTC, PMTCT, prevention of mother-to-child transmission; ARVs, antiretrovirals; NVP nevirapine; Unit costs in 2013 US Dollars

^a^Weighted mean represents a nationally representative average value, taking into account the relative contribution of each facility in terms of its patient volume. It was calculated as the summation of each data point multiplied by a non-negative weight (defined as the number of annual HTC clients/Outpatient health clients). Therefore, data points with a higher weight contribute more to the weighted mean than do elements with a low weight.
